# Characterization of a miRNA Signature with Enhanced Diagnostic and Prognostic Power for Patients with Bladder Carcinoma

**DOI:** 10.3390/ijms242216243

**Published:** 2023-11-13

**Authors:** Maria Samara, Panagiotis J. Vlachostergios, Eleni Thodou, Ioannis Zachos, Lampros Mitrakas, Konstantinos Evmorfopoulos, Vassilios Tzortzis, Antonis Giakountis

**Affiliations:** 1 Department of Pathology, Faculty of Medicine, School of Health Sciences, University of Thessaly, 41335 Larissa, Greece; 2 Department of Medicine, Division of Hematology and Medical Oncology, Weill Cornell Medicine, New York, NY 10065, USA; 3 Department of Urology, Faculty of Medicine, School of Health Sciences, University of Thessaly, University Hospital of Larissa, 41335 Larissa, Greece; 4 Department of Biochemistry and Biotechnology, University of Thessaly, 41335 Larissa, Greece

**Keywords:** miRNAs, bladder carcinoma, diagnosis and prognosis, genitourinary transcriptomics, ROC analysis

## Abstract

Bladder carcinoma is globally among the most prevalent cancers and is associated with a high mortality rate at advanced stages. Its detection relies on invasive diagnostic methods that are unpleasant for the patient. Non-invasive molecular biomarkers, such as miRNAs, could serve as alternatives for early detection and prognosis of this malignancy. We designed a computational approach that combines transcriptome profiling, survival analyses, and calculation of diagnostic power in order to isolate miRNA signatures with high diagnostic and prognostic utility. Our analysis of TCGA-BLCA data from 429 patients yielded one miRNA signature, consisting of five upregulated and three downregulated miRNAs with cumulative diagnostic power that outperforms current diagnostic methods. The same miRNAs have a strong prognostic significance since their expression is associated with the overall survival of bladder cancer patients. We evaluated the expression of this signature in 19 solid cancer types, supporting its unique diagnostic utility for bladder carcinoma. We provide computational evidence regarding the functional implications of this miRNA signature in cell cycle regulation, demonstrating its abundance in body fluids, including peripheral blood and urine. Our study characterized a novel miRNA signature with the potential to serve as a non-invasive method for bladder cancer diagnosis and prognosis.

## 1. Introduction

Urothelial bladder carcinoma (BLCA) ranks as the seventh most frequently diagnosed cancer among males but falls to the tenth position when both genders are included. The incidence and mortality rates of bladder cancer exhibit variations among different countries due to disparities in risk factors, practices related to detection and diagnosis, and accessibility of treatment options [1]. The majority of BLCA are non-muscle-invasive carcinomas with a high risk of recurrence within the first year after diagnosis [2,3]. Non-invasive traditional methods such as ultrasound scans and cytology evaluation are widely accepted methods for making a diagnosis, as both are very easy and cost-effective procedures; however, their sensitivity, specificity, and repeatability are to some extent limited [4]. To date, cystoscopy is generally considered the gold standard for BLCA diagnosis. However, due to its invasive nature and associated discomfort, there is a pressing need to explore alternative non-invasive procedures for the identification of novel biomarkers.

Non-invasive molecular approaches based on the assessment of protein biomarkers have been proposed as alternatives to invasive diagnostic procedures, provided that their sensitivity exceeds 90% [5]. ELISA-based techniques have been widely used for the detection of various antigens, such as the NMP22 chromatin regulator, complement factor H-related protein, or bladder tumor antigen (BTA), with variable sensitivity and sensitivity rates [6,7]. Immunofluorescence and fluorescence in situ hybridization (FISH) techniques, such as ImmunoCyt and Urovision, detecting either abnormal proteins or chromosomal aberrations, have also been used for the diagnosis and follow-up of BLCA [8,9]. Beyond antigens, other tests, such as UroSEEK, aim at detecting tumor DNA or even direct DNA methylation (EpiCheck) as an epigenetic marker for bladder cancer [10,11].

Nowadays, despite the existence of several non-invasive urine biomarkers that have been established for the purpose of diagnosing urothelial cancer, it is worth noting that urine cytology stands as the sole liquid biopsy method indicated for the surveillance of BLCA in several treatment guidelines [12]. Urinary cytology is commonly used in routine practice due to its high specificity in the diagnosis of high-grade urothelial carcinoma, though it shows a low sensitivity for low-grade urothelial carcinoma [13,14,15].

Given the expanding knowledge of liquid biopsy in the field of oncology, many studies have focused on the introduction of new biomarkers in diverse biological fluids, such as blood, plasma, urine, cerebrospinal fluid, and saliva, not only for monitoring cancer progression but also for diagnosis [16,17]. In this context, microRNAs (miRNAs), a diverse group of short non-coding RNAs of 20-22 nucleotides in length, may serve as helpful biomarkers of diagnostic and prognostic value in various human diseases [18,19]. A miRNA molecule is capable of targeting and controlling hundreds of genes implicated in variable biological processes [20,21]. Bioinformatic analysis has revealed that a range of 30% to 60% of human coding genes have the potential to be regulated by miRNAs [20]. miRNAs could suppress the expression of their target genes by promoting the breakdown of mRNAs (messenger RNAs) or by interfering with their translation process. Therefore, they play a crucial role in the diagnosis and prognosis of various conditions, depending on their classification as tumor suppressors (TSs) or oncogenic [22].

The precise mechanisms by which miRNAs are altered in the bladder are still poorly understood. Previous studies have shown that miRNAs in bladder cancer are dysregulated, aiding cell proliferation and epithelial–mesenchymal transition (EMT) and inhibiting apoptosis [23]. The dysregulation of miRNAs is strongly associated with multiple biological processes, including cell cycle arrest, apoptosis, proliferation, metastasis, treatment resistance, and other activities. Numerous miRNAs have been associated with tumor type, stage, or patient survival. Due to their notable stability in biological fluids, miRNAs are recognized as a promising group of biomarkers for the early detection of many human malignancies, including bladder cancer [24]. To date, many miRNA signatures have been identified, some of which have superior diagnostic efficacy in comparison to others. However, there is no consensus on a definite diagnostic miRNA signature, emphasizing the necessity for further research [25].

Below, we present the results of a computational analysis that relies on publicly available TCGA data to evaluate miRNA expression in terms of diagnostic and prognostic performance for bladder cancer diagnosis. Our analysis provided a molecular signature consisting of eight miRNAs that collectively outperform existing diagnostic methods. This miRNA signature complements existing diagnostic approaches, facilitating the development of molecular biomarkers for the non-invasive detection of bladder cancer.

## 2. Results

### 2.1. miRNA Differential Expression Analysis in TCGA Bladder Cancer Biopsies

**Figure 1:**
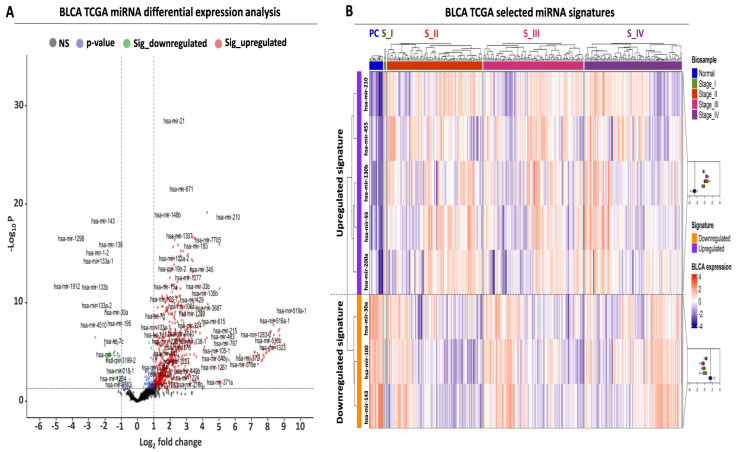
Differential expression analysis of miRNAs in TCGA-BLCA patient cohort. (**A**) Volcano plot analysis highlighting differentially expressed miRNAs (DEMs) between BLCA tumors (N = 409) and paracancerous tissues (N = 19). Green and red dots correspond to significantly down- and upregulated miRNAs, respectively. Vertical dashed lines indicate the log2FC thresholds (log2FC < −1 or > 1), while the horizontal dashed line highlights the statistical threshold (transformed − log10 *p*-value > 1.3, corresponding to *p*-value < 0.05). (**B**) Heatmap analysis highlighting the expression of the selected miRNAs in BLCA tumors. Tumors are stratified according to the pathological stage and are color-coded accordingly at the top horizontal annotation of the heatmap. The selected miRNAs are divided and color-coded as up- (purple) or downregulated (orange), as indicated in the left-row annotation. Box plots, shown on the right, illustrate the expression of up- or downregulated miRNAs in all tumor stages against that of miRNAs in paracancerous (normal) biopsies, the average expression of which is highlighted with the vertical dashed line inside the box plot. Heatmap colors correspond to Z-scores of normalized miRNA expression.

Transcriptome analysis was performed in BLCA biopsies from the TCGA consortium with the aim of isolating differentially expressed miRNAs with an elevated diagnostic and prognostic potential, using three selection criteria. First, comparative analysis between tumor and paracancerous biopsies revealed a total number of 794 differentially expressed miRNAs (DEMs), corresponding to 56.7% of all analyzed miRNAs (Figure 1A, Supplementary Table S1). Based on standard cutoffs for differential expression (see M&M, Section 4.2), the majority of these DEMs were upregulated (756 or 54% of analyzed miRNAs), while only 38 miRNAs were significantly downregulated (2.7% of analyzed miRNAs).

Apart from the observed differences in absolute miRNA numbers, an overall stronger pattern of upregulation was also observed since the fold change range of the upregulated miRNAs was two times higher compared to the downregulated miRNAs (upregulated log2 fold change (FC) range: 1 to 9, downregulated log2FC range: −1 to −4, Figure 1A, Supplementary Table S1). Taken together, these data support the existence of a large number of DEMs between BLCA tumors and paracancerous biopsies, with an observed bias toward upregulation in tumor samples.

### 2.2. Establishment of a miRNA Signature for BLCA Diagnosis

Having detected several DEMs for BLCA, we subsequently focused on the establishment of a miRNA signature that is suitable for early-stage detection. Among our top DEMs, we selected a small number of miRNAs, the expression of which was significantly altered between early and late BLCA tumor stages compared to paracancerous levels (Figure 1B, Supplementary Table S2). Therefore, differential expression in each tumor stage compared to paracancerous expression levels served as a second criterion for selecting each of the shortlisted DEMs, applying the same criteria as described in Section 4.2 as minimum cutoffs. More specifically, the upregulated miRNA signature consisting of five miRNAs (hsa-mir-210, hsa-mir-455, hsa-mir-130b, hsa-mir-93, and hsa-mir-200a) was significantly overexpressed in tumors of all American Joint Committee on Cancer (AJCC) tumor stages (I–IV) compared to paracancerous tissue. The average expression change of this miRNA signature ranged between 7.9- and 11.9-fold (log2FC mean difference: 2.98–3.57), and its upregulation pattern was consistent among the four tumor stages compared to their presence in paracancerous tissues (Supplementary Table S2).

Moreover, we complemented the upregulated miRNA signature with three miRNAs (hsa-mir-30a, hsa-mir-100, and hsa-mir-143) that were significantly downregulated in all tumor stages compared to paracancerous tissue levels (Figure 1B, Supplementary Table S2). Similar to the observations on the upregulated miRNAs, the downregulation of the three selected DEMs was consistent in tumor tissue biopsies ranging between 0.12- and 0.22-fold (log2FC mean difference: −2.17 and −3.1) (Supplementary Table S2). In conclusion, our differential expression analysis highlighted an 8-miRNA signature consisting of five upregulated and three downregulated DEMs, the expression of which was significantly and consistently altered even from the early stages of BLCA compared to their paracancerous counterpart.

### 2.3. Evaluation of the Diagnostic and Prognostic Value of the Upregulated miRNA Signature

Differential expression analysis is useful in isolating DEMs with altered levels between cancerous and non-cancerous tissues, but these changes are not always indicative of an increased diagnostic potential for the respective miRNAs. We, therefore, performed a receiver operating curve (ROC) analysis with the aim of evaluating the performance of our miRNA signatures in differentiating between BLCA tumor and paracancerous tissues and using it as a third miRNA selection criterion. We applied an ROC AUC (area under curve) cutoff of 0.8 as a minimum threshold for selecting the final miRNA signatures.

**Figure 2:**
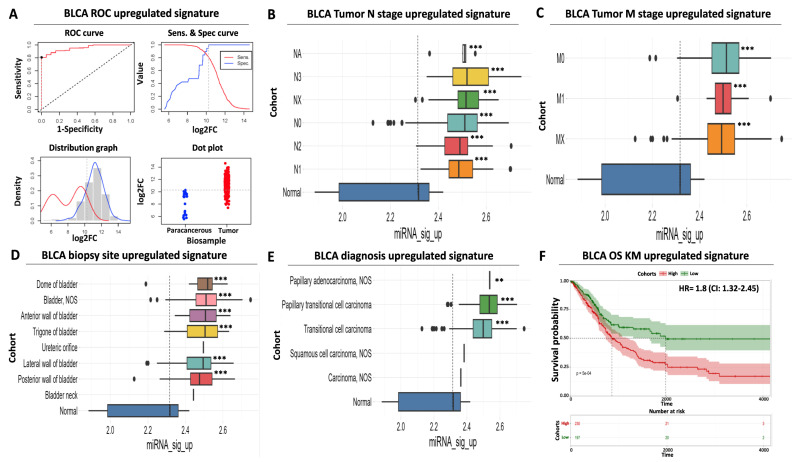
Diagnostic and prognostic significance of the upregulated miRNA signature. (**A**) ROC analysis for discriminating BLCA tumor vs. paracancerous tissues. ROC curve (upper left) indicates an increased diagnostic potential (AUC = 0.95) of the upregulated miRNA signature shown in red. The cutoff analysis curve (upper right) indicates the optimal expression cutoff point (10.26) for separating tumors from paracancerous biopsies. The bottom left distribution graph highlights the expression range of the miRNAs that constitute the upregulated signature in paracancerous (red) or tumor (blue) samples. Finally, the bottom right dot plot highlights the average expression of the upregulated miRNA signature in individual paracancerous (blue) or tumor (red) biopsies. The horizontal dashed line indicates the calculated optimal cutoff point. (**B**) Box plot analysis indicating the elevated expression of the upregulated miRNA signature according to regional lymph node status of AJCC tumor invasion compared to paracancerous levels. (**C**) Same as in (**B**) but for AJCC distant metastasis status. (**D**) Box plot analysis highlighting the elevated expression of the upregulated miRNA signature in various anatomical/histological sites of BLCA biopsies within the bladder. (**E**) Box plot analysis depicting the elevated levels of the upregulated signature in common histological variants of BLCA. The vertical dashed line in all box plots marks average miRNA expression in paracancerous samples. (**F**) Kaplan–Meier analysis showing a statistically significant association between elevated expression of the upregulated miRNA signature (red curve) and shorter overall survival time of BLCA patients. Dashed lines indicate duration for 50% probability of survival since diagnosis. Asterisks in all graphs indicate the level of statistical significance based on ANOVA (***: *p*-value < 0.001, **: *p*-value < 0.01).

ROC analysis indicated that our selected five DEMs that constitute the upregulated miRNA signature are characterized by a robust and statistically significant sensitivity in differentiating tumors from paracancerous samples (Figure 2A). More specifically, the individual AUC performance of these DEMs ranged from 0.95 to 0.82, while the AUC of the combined upregulated signature was near 0.96 (CI: 0.92–0.98), with an optimal cutoff point of expression that equals 10.26 on a log2 scale (Supplementary Table S3).

Surprisingly, although the expression of the upregulated DEMs follows the binomial pattern in tumor samples, their expression in paracancerous biopsies follows a bimodal pattern of expression (Figure 2A). It can be postulated that this may be due to differences in the proximity of the paracancerous tissue to the tumor or due to physiological factors that alter DEM levels. Nevertheless, our calculated cutoff considers this observed variability to minimize the probability of false positive detection. Importantly, the AUC performance of other top upregulated miRNAs from our differential expression analysis (Supplementary Table S1) was inferior compared to our selected DEMs (Supplementary Table S3), again supporting the necessity of evaluating diagnostic performance using a combination of ROC and differential expression analysis.

In addition to the above, a significantly elevated expression of the same DEMs was uniformly observed regardless of patient clinical characteristics, including tumor nodal status (ranging between 7.2- and 12.4-fold higher than average paracancerous levels, Figure 2B, Supplementary Table S2) or presence of distant metastases (ranging between 7.8 and 9.4-fold higher than average paracancerous levels, Figure 2C, Supplementary Table S2). This finding is concordant with the previously observed increase in average signature levels in all tumor stages compared to precancerous tissues (Figure 1B). We also confirmed that the average expression of our upregulated miRNA signature is significantly increased in all common biopsy sites (ranging between 5.4- and 9.5-fold higher than average precancerous levels, Figure 2D, Supplementary Table S2) or BLCA histological variants (ranging between 2.9- and 11.1-fold higher than average precancerous levels, Figure 2E, Supplementary Table S2), supporting its suitability for robust and versatile diagnosis of BLCA.

Further to its elevated diagnostic potential, our upregulated miRNA signature is also significantly associated with poor prognosis. More specifically, Kaplan–Meier analysis revealed that elevated mean levels of the upregulated signature are associated with a significantly shorter overall survival time of BLCA patients (50% survival probability of 900 days for the high-expressing cohort against 1.950 days for the low-expressing patient cohort), while elevated expression in tumors is associated with a hazard ratio (HR) of 1.8 (95% confidence interval (CI): 1.3–2.4, Figure 2F). Taken together, these data highlight the significant diagnostic and prognostic value of our upregulated miRNA signature regardless of tumor stage, histological variant, or biopsy site.

### 2.4. Evaluation of the Diagnostic and Prognostic Value for the Downregulated miRNA Signature

Despite the robust diagnostic and prognostic performance of our upregulated miRNA signature, its sole application cannot exclude the misidentification of false positives or negatives among BLCA biopsies, thus limiting its clinical application as a molecular biomarker. To eliminate the possibility of such ambiguities, we also evaluated the diagnostic and prognostic utility of our downregulated miRNA signature with the aim of using it in conjunction with our upregulated DEMs.

**Figure 3:**
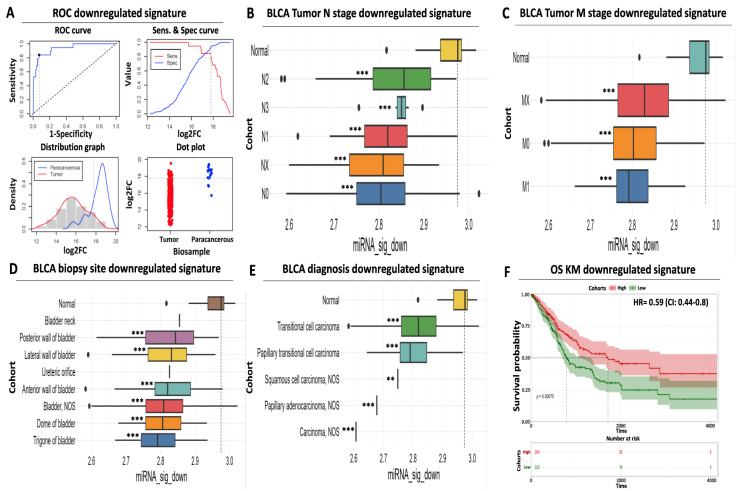
Prognostic and diagnostic significance of the downregulated miRNA signature (**A**) ROC analysis for discriminating paracancerous vs. tumor BLCA biopsies. ROC curve (upper left) indicates an increased diagnostic potential (AUC = 0.93) of the downregulated miRNA signature shown in blue. Cutoff analysis (upper right) indicates the optimal expression cutoff point (17.79) for separating paracancerous from tumor biopsies. The bottom left distribution graph highlights the expression range of the miRNAs that constitute the downregulated signature in paracancerous (blue) or tumor (red) samples. Finally, the bottom right dot plot highlights the average expression of the downregulated miRNA signature in individual paracancerous (blue) from tumor (red) biopsies. The horizontal dashed line indicates the calculated optimal cutoff point. (**B**) Box plot analysis indicating the elevated expression of the downregulated miRNA signature in paracancerous samples compared to regional lymph node status. (**C**) Same as in (**B**) according to the presence of distant metastasis. (**D**) Box plot analysis highlighting the uniformly decreased expression of the downregulated miRNA signature in various anatomical sites of BLCA biopsies. (**E**) Box plot analysis depicting the decreased levels of the downregulated signature in histological variants of BLCA. The horizontal dashed line marks the average miRNA expression in paracancerous samples in all box plots. (**F**) Kaplan–Meier analysis depicting a statistically significant association between reduced mean expression (green curve) of the downregulated miRNA signature and reduced overall survival of BLCA patients. Dashed lines indicate the duration for a 50% probability of survival. Asterisks in all graphs indicate the level of statistical significance based on ANOVA (***: *p*-value < 0.001, **: *p*-value < 0.01).

Similar to our upregulated miRNA results, ROC analysis confirmed the increased diagnostic significance of our downregulated DEMs, with a calculated AUC for the individual miRNAs ranging between 0.86 and 0.9 (Figure 3A, Supplementary Table S4). The AUC performance of the combined downregulated signature was further increased to 0.93 (CI: 0.88–0.99), suggesting that utilization of a limited number of carefully selected miRNAs can increase diagnostic accuracy compared to individual miRNA performance. Our cutoff analysis indicates 17.79 as an optimal log2 expression threshold for discriminating paracancerous tissue from tumor samples. This threshold is 185-fold higher than the calculated cutoff for the upregulated signature, ensuring significant separation and, therefore, robust differentiation of the biological samples. Interestingly, we did not observe a bimodal distribution for the expression of our downregulated miRNA signature expression since both the paracancerous and the tumor histograms follow the binomial distribution (Figure 3A), facilitating sharp biological sample identification.

Next, we assessed the expression of our downregulated DEM signature according to various clinico-pathological characteristics. Following the opposite trend compared to the upregulated signature, the mean expression of our downregulated miRNAs was significantly reduced across tumors of different nodal status (ranging between 4 and 26% of average paracancerous levels, Figure 3B, Supplementary Table S2) or distant metastasis (ranging between 15 and 20%, Figure 3C, Supplementary Table S2). We confirmed that this downregulation pattern in the tumors is maintained among most common biopsy sites (ranging between 15 and 27% of average paracancerous levels, Figure 3D, Supplementary Table S2) and all common diagnostic BLCA subtypes (ranging between 2 and 18% of average paracancerous levels, Figure 3E, Supplementary Table S2), supporting its potential as a diagnostic tool for all stages of BLCA.

Apart from its diagnostic value, our downregulated signature also holds a prognostic utility. Kaplan–Meier analysis revealed a statistically significant association between low mean expression levels of downregulated miRNAs in tumors and reduced survival of BLCA cancer patients (50% survival probability of 800 days for the low-expressing cohort against 1.650 days for the high-expressing cohort, Figure 3F). In addition, elevated expression of the same miRNAs in tumors was associated with an HR of 0.59 (95% CI: 0.44–0.8, Figure 3F), highlighting the protective function of this signature against bladder cancer. In conclusion, these results support the diagnostic and prognostic potential of our downregulated miRNA signature, the utilization of which complements the performance of our upregulated miRNA signature for early detection of bladder carcinoma.

### 2.5. Multicancer Comparisons of the Selected miRNAs

Having established two selective miRNA signatures with high predictive power for BLCA diagnosis and prognosis, our next goal was to assess whether this is a BLCA-specific effect or whether their expression pattern across multiple cancer types might play a similar role in diagnosis and/or prognosis. Thus, we examined the selectivity of each signature for BLCA against other genitourinary malignancies and various primaries whereby the same miRNAs are differentially expressed and, therefore, could potentially serve as diagnostic biomarkers. We also included a third signature of eight randomly selected miRNAs to serve as control in the analysis.

**Figure 4:**
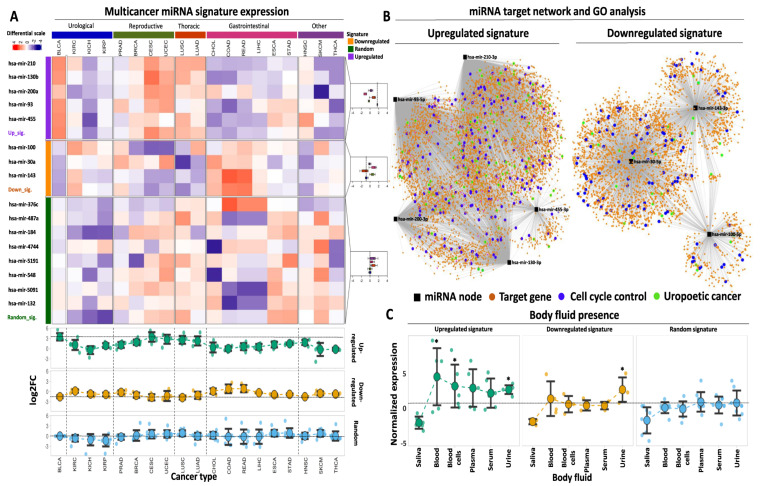
Multicancer and body fluid expression analysis of the miRNA signatures. (**A**) Heatmap illustrating the expression of the up- and downregulated miRNAs in 19 cancer types from TCGA against a randomly selected miRNA signature of equal size. Tumor types are broadly annotated at the top of the heatmap according to their anatomical primary sites. The selected miRNAs are divided and color-coded as upregulated (purple), downregulated (orange), or randomly expressed (green). as indicated in the left-row annotation. Box plots, shown on the right, indicate the expression of up-, downregulated, or randomly expressed miRNAs in different primaries, the mean expression of which is highlighted with the vertical dashed line inside the box plot. Heatmap colors correspond to Z-scores of normalized miRNA expression. The bottom dot plots indicate average log2FC levels of each signature (upregulated, downregulated, or random) across the 19 analyzed cancer types. Horizontal dashed lines indicate the average expression of each signature in BLCA. An explanation of tumor type abbreviations is available at https://gdc.cancer.gov/resources-tcga-users/tcga-code-tables/tcga-study-abbreviations (accessed on 1 October 2023)). (**B**) miRNA target network and GO analysis for both miRNA signatures. Black square nodes correspond to the five miRNAs that constitute the upregulated signature (shown on the left) or the three miRNAs that constitute the downregulated signature (shown on the right). Predicted mRNA targets are shown with the orange circle nodes and are connected with their respective miRNA regulator(s) with gray edges. Blue circles highlight mRNA target genes that are involved in cell cycle regulation, while green circles highlight target genes that are associated with BLCA or genitourinary neoplasms in general. (**C**) Dot plots demonstrating average normalized expression of the up-, downregulated, or random miRNA signatures in various body fluids. Data represent mean ± S.E. The dashed horizontal line highlights the mean expression of the random miRNA signature in urine. Asterisks highlight statistical significance compared to the random miRNA signature (*: *p*-value < 0.05).

Our multicancer survey revealed that the mean expression fold change of the upregulated signature in BLCA outperformed all other types of cancer, excluding cervical squamous cell carcinoma (CESC) in which log2 fold change between tumor and paracancerous samples was comparable to the one for BLCA (94.5% of BLCA outperformance among the analyzed tumor types, Figure 4A). With respect to individual miRNAs, the fold change of hsa-mir-200a in CESC and uterine corpus endometrial carcinoma (UCEC) exceeded that of BLCA, which in turn outperformed all other cancers (89% of BLCA outperformance). In addition, hsa-mir-130b also showed stronger differential expression in CESC compared to BLCA, but the BLCA fold change outperformed all other types of cancer (94.5% of BLCA outperformance). The fold change of tumor vs. paracancerous expression for each of the remaining upregulated miRNAs was higher in BLCA compared to all other cancer types that were tested.

Comparing the performance of our upregulated signature against a randomly selected collection of miRNAs, an 11-fold higher mean expression was found compared to the mean levels of the randomly selected miRNAs (Supplementary Table S5). When the same signature was tested across other tumor types, its expression remained statistically higher compared to the random signature in three out of five groups of malignancies (namely urological, reproductive, and thoracic) with 4-fold higher expression compared to the random signature (Supplementary Table S5).

With regards to the performance of the downregulated signature across different tumor types, only two types of malignancies (CESC characterized by 31% downregulation and lung squamous cell carcinoma—LUSC with 29% downregulation of the signature in the tumors) outperformed BLCA that was associated with 34% downregulation. The expression of the downregulated signature in all other cancer types was higher compared to BLCA (89% of BLCA outperformance). With respect to individually downregulated DEMs, hsa-mir-100 showed similar or more pronounced downregulation in five cancer types compared to BLCA (72.2% of BLCA outperformance), while hsa-mir-30a and hsa-mir-143 showed similar or more pronounced downregulation in three and two other cancer types, respectively, compared to BLCA (83.3% and 89% of BLCA outperformance). Among these cancer types, reproductive (breast carcinoma—BRCA, prostate adenocarcinoma—PRAD, CESC, and UCEC), together with thoracic-related malignancies, were overrepresented.

As expected, our statistical analysis revealed that expression of our downregulated miRNA signature was significantly lower in BLCA tumors compared to the levels of the randomly selected miRNAs (Supplementary Table S5). When assessing different primaries, the difference between the downregulated and the random signature was significant for three out of five malignancies, yet in contrast to the upregulated signature, the same comparison was not significant for urological cancers, including BLCA and kidney cancer. Of note, the mean expression of both signatures in BLCA outperformed the expression in three types of kidney cancer (kidney chromophobe—KICH, kidney renal clear cell—KIRC, and kidney renal papillary cell carcinoma—KIRP), further supporting their specificity for BLCA. Collectively, our multicancer comparison approach underlines the potent tumor-specific properties of the selected miRNA signatures for tumor versus paracancerous biopsies, specifically for bladder cancer, as compared to the majority of the most common types of solid tumors.

### 2.6. Target Network and GO Analysis of Selected miRNAs

miRNAs exert their physiological or aberrant function through the regulation of downstream target genes at the post-transcriptional or translational level. With the aim of providing a first line of functional evidence regarding the regulatory implications of our selected miRNA signatures in BLCA, we performed target network construction followed by gene ontology (GO) analysis separately for each miRNA signature.

This approach revealed an extensive network of interactions that are governed by the five miRNAs that constitute the upregulated signature (Figure 4B). Among them, hsa-mir-210 and 93 were predicted to affect the highest number of mRNA targets, followed by hsa-mir-200 and 130b, while hsa-mir-455 had the smallest number of predicted targets. miRNA disease enrichment analysis for the same DEMs revealed their significant association with various cancer types, including BLCA (Supplementary Table S6A). In addition, disease gene ontology (GO) enrichment of their mRNA targets again revealed significant associations with several malignancies (Supplementary Table S6B). These affected mRNAs are strongly and statistically associated with various aspects of cell cycle regulation (Figure 4B, Supplementary Table S6C), underlining their functional involvement not only in BLCA but also in other malignancies.

Target network analysis for the DEMs that constitute the downregulated signature revealed several mRNAs that are potentially under the control of these miRNAs. hsa-mir-30a had the highest number of predicted target mRNAs, followed by hsa-mir-143 and hsa-mir-100 (Figure 4B). All three miRNAs were predicted to be involved in various malignancies, including urothelial carcinoma, complementing the observations of the upregulated miRNAs. At the mRNA target level, disease enrichment analysis was concordant with the results of the upregulated signature, confirming the involvement of the predicted targets in various malignancies, including BLCA. Finally, GO analysis of the mRNA targets highlighted their multidimensional involvement in cell proliferation and regulation of the cell cycle (Supplementary Table S7C). In conclusion, these results support the notion that the selected miRNA signatures constitute a regulatory network that affects the expression and function of multiple mRNA targets with significant implications for cell cycle regulation and cancer progression.

### 2.7. miRNA Presence in Body Fluids

One crucial property of miRNAs is their presence in various body fluids, which facilitates their utilization as non-invasive biomarkers for early and cost-effective cancer detection. In order to explore the non-invasive potential of our selected miRNAs, we assessed their expression in publicly available miRNA transcriptome data in various human body fluids. We found that our upregulated miRNA signature is significantly overexpressed in whole blood and urine compared to the mean expression of the randomly selected miRNAs in the same body fluids (Figure 4C, Supplementary Table S8). Moreover, expression of the same miRNAs is elevated in serum and plasma compared to the random miRNAs, albeit not statistically different due to the small sample size. Regarding the circulating nature of our downregulated miRNAs, their expression was significantly increased in urine compared to the random signature, with no significant alterations in the remaining body fluids based on this analysis (Figure 4C, Supplementary Table S8). However, these transcriptomes represent body fluids from cancer patients and, therefore, largely underestimate the expression of our tumor-downregulated DEMs. In support of this hypothesis, the expression of our tumor-upregulated signature was found to be increased in most body fluids compared to the expression of the downregulated signature (Figure 4C). Of note, both miRNA signatures were not present in saliva, similar to the randomly selected miRNAs, suggesting that this body fluid potentially lacks miRNA that could be used for BLCA diagnosis. It should be noted that in the lack of body fluid data from healthy individuals, we cannot confirm or reject the differential expression of our selected miRNAs at the body fluid level between cancer patients and healthy donors. Nevertheless, the data above support the presence of our miRNA signatures in patient body fluids, supporting an initial potential for non-invasive detection. It is within our short-term plans to experimentally test our miRNA signatures for differential expression between healthy and patient body fluids, testing their efficiency as non-invasive diagnostic biomarkers.

## 3. Discussion 

To date, bladder cancer diagnosis largely relies on invasive methods such as cystoscopy-based biopsy, which is unpleasant for patients and can confound accurate detection of some subtypes of this malignancy, especially in the early stages of the disease [26]. Non-invasive traditional methods such as urinary cytology have been ancillary to cystoscopy in clinical practice; however, their low sensitivity, which typically ranges below 40%, limits their utility as alternative diagnostic tools [27]. Due to their regulatory role and expression properties [28], miRNAs have long been proposed to serve as diagnostic and/or prognostic markers in pathology [29], including infectious [30,31] and non-infectious diseases [32,33], including various types of cancer [34,35].

Several miRNAs have been previously proposed as non-invasive biomarkers for bladder cancer [36]. Recently, a 4-miRNA panel consisting of hsa-miR-182-5p, hsa-miR-196a-5p, hsa-miR-124-3p, and hsa-miR-34a-5p exhibited a sensitivity of 98%, specificity of 93% and an AUC of 0.98 (CI: 0.952 to 0.998, *p*-value < 0.001) [37]. However, the performance of the same 4-miRNA signature in TCGA-BLCA data was poorer, with a sensitivity of 0.77, specificity of 0.84, and AUC of 0.69 (CI: 0.55–0.84, Supplementary Table S9). The performance of our miRNA signatures in TCGA-BLCA data was superior to the reported 4-miRNA signature with an AUC of 0.95 (CI: 0.92–0.98, *p*-value < 0.001) for the upregulated and 0.93 (CI: 0.88–0.99, *p*-value < 0.001) for the downregulated DEMs, along with improved specificities and sensitivities. This lower diagnostic power and accuracy of the published miRNAs in the TCGA cohort could in part be attributed to their original detection via RT-qPCR in serum, while TCGA-BLCA data are generated through miRNA sequencing in solid biopsies. In addition, preliminary evidence suggests that our selected miRNAs are present in urine, and their BLCA performance, in terms of expression ratio in tumor vs. paracancerous tissues, outperforms a large number of solid tumor types, properties that were not tested for this recently published miRNA signature.

Another recent publication utilized TCGA-BLCA data to evaluate the diagnostic and prognostic performance of ten prioritized miRNAs [38]. According to these results, the diagnostic AUC of the ten miRNAs ranged between 0.53 and 0.82. The poor AUC performance that is observed for some of these miRNAs agrees with their reported lack of significance for differential expression between BLCA tumors and paracancerous tissues [38]. We retained only DEMs in our initial selection round of transcriptome analysis and subsequently excluded all DEMs with AUC less than 0.80, resulting in an AUC range between 0.82 and 0.96 for our chosen miRNAs. In terms of prognostic power, the expression of the final six selected miRNAs from the same report has been significantly associated with overall survival. This prognostic performance is comparable to the reported one for the 6-miRNA signature [38] or of the 21 miRNAs that were also analyzed previously [39]. Again, none of these studies tested the expression of these miRNAs in other tumor types compared to BLCA. Of note, some of our final DEMs are among those reported 21 miRNAs [39], suggesting that our stringent selection coupled with the cumulative signature analysis not only retains diagnostic and prognostic performance but also simplifies detection over a large panel of miRNAs.

In terms of target prediction and function, our miRNA network and GO analysis suggest that both signatures regulate target networks that, in turn, govern critical cancer-related aspects of tumor biology, such as cell cycle. For example, hsa-mir-200a, which is among our selected miRNAs, has been reported to regulate the cell cycle by targeting the tumor necrosis factor α-induced protein 3 (A20, [40]). hsa-mir-210 is regulated by HIF and acts as a critical link between hypoxia and cell cycle regulation in cancer [41,42]. hsa-mir-93 has been associated with poor prognosis in pancreatic cancer [43] and is reported to target LATS2, which encodes for a tumor suppressor kinase, ultimately enhancing metastasis [44]. Its presence in exosomes from bladder cancer patients has been reported as an important determinant for the progression of the disease [45]. Finally, hsa-mir-130b also promotes the proliferation of bladder cancer cells via targeting of VGLL4 [46].

With regards to the downregulated miRNAs, hsa-mir-100 has been negatively correlated with the protein levels of FGFR3, a critical regulator in many forms of cancer [47]. hsa-mir-30a has been reported to function as an inhibitor of cell proliferation and/or disease progression in many cancer types [48], including but not limited to bladder [49], liver [50], and glioma [51]. Finally, hsa-mir-143 has been shown to inhibit proliferation of bladder cancer cells through repression of the IGF-1R cascade [52]. Taken together, these reports not only support our GO results regarding the critical function of our selected DEM in bladder tumors or cancer in general but also provide a functional basis for the observed differential expression of some selected miRNAs in other tumor types.

Our study is limited by its computational design without in vitro validation, which, however, represents a strong hypothesis-generating analysis that may pave the way for further investigations. These results could substantially help evaluate the full potential of both miRNA signatures for early and non-invasive detection and prognosis of BLCA, as well as elucidate the molecular mechanisms accounting for their function in bladder tumors.

## 4. Materials and Methods

### 4.1. TCGA Data Acquisition and Preprocessing

Data from the Cancer Genome Atlas program (TCGA) were downloaded as level 3 miRNA expression values from the GDC Data Portal (https://gdc.cancer.gov/ (accessed on 17 July 2023)), along with their clinical information. The TCGA-BLCA miRNA dataset consists of 429 samples, subdivided into 410 tumor and 19 matched paracancerous samples. No significant biases were observed for the paracancerous biopsies regarding the tumor stage of their matched tumor samples, with distributions ranging around 25%, indicating random distribution across all tumor stages. Prior to the analysis, the raw expression data of 1870 miRNAs were subjected to filtering to remove those with mean expression below the 25% percentile across all samples, resulting in the removal of 471 low or not-expressed miRNAs in the BLCA dataset (25% of total). The remaining 1399 miRNAs were subsequently subjected to downstream analysis, as indicated below. ANOVA analysis did not observe any bias regarding the preference of these miRNAs for a particular tumor stage, with no statistically significant changes in average miRNA between tumor stages, suggesting their even distribution. 

### 4.2. miRNA Differential Expression Analysis

The expression data of the filtered miRNAs were subjected to differential expression analysis in paracancerous vs. tumor biopsies with the Bioconductor package edgeR (https://bioconductor.org/packages/release/bioc/html/edgeR.html (accessed on 20 July 2023) [53,54,55]) in R. The applied thresholds for differential expression analysis were false discovery rate (FDR) < 0.05 and log2FC > 1 or < −1 for tumor-upregulated or tumor-downregulated miRNAs, respectively.

Volcano plots were prepared with the EnhancedVolcano Bioconductor package in R (https://bioconductor.org/packages/release/bioc/html/EnhancedVolcano.html (accessed on 22 July 2023) [56]).

### 4.3. Heatmap Construction of miRNA Signature Expression in TCGA-BLCA Patient Data

miRNA expression quantifications for the final 8-miRNA signature in the complete TCGA-BLCA patient cohort were log2 transformed and converted to z-scores in R. Patient samples were annotated as paracancerous or tumor, the latter of which were further stratified as stage I, II, III, and IV based on the available clinical information from the TCGA platform. The Bioconductor ComplexHeatmap package [57] was used to construct separate heatmaps in R for the 5-miRNA upregulated signature, the 3-miRNA downregulated signature, or the 8 randomly selected miRNAs that served as control. ComplexHeatmap arguments included row and column clustering based on the Pearson coefficient, together with column split according to biosample annotation and row split and grid boxplot construction according to miRNA signature annotation.

### 4.4. ROC Analysis of Selected miRNA Signatures

The selected miRNAs from the differential expression analysis were subjected to ROC calculations with the Github package EasyROC [58], while a cutoff analysis was performed with the Github package OptimalCutpoint [59] in R. The selection criteria for elevated diagnostic power of the selected miRNAs were *p*-value < 0.05 and AUC ≥ 0.8. All ROC plots were generated in R.

### 4.5. Box Plot and Statistical Analysis of miRNA Expression According to Patient Clinic-Pathological Characteristics in Multiple Cancer Types

miRNA expression was compared against four patient clinical and pathological characteristics, namely, AJCC tumor stages I to IV, AJCC regional lymph node status N0-Nx, AJCC distant organ metastasis M0-Mx, BLCA biopsy site, and BLCA primary diagnosis, according to TCGA-BLCA clinical information. A normality test was performed with Shapiro prior to ANOVA Holm–Sidak and multiple comparisons test, all performed with SigmaPlot 11 https://alfasoft.com/software/statistics-and-data-analysis/data-visulization/sigmaplot/ (accessed on 25 July 2023)). All box and dot plots were prepared with ggplot2 (https://ggplot2.tidyverse.org/ (accessed on 29 July 2023)) in R.

### 4.6. Network and GO Analysis

For the miRNA network, the miRbase IDs of the final miRNA signature were used as input for network construction using miRNET [60], focusing on the analysis of miRTarBase v8 genes. Functional analysis was performed with KEGG and DisGeNET through hypergeometric enrichment.

### 4.7. Kaplan–Meier Analysis of miRNA Signatures

For the Kaplan–Meier analysis, miRNA expression and patient clinical characteristics from the TCGA-BLCA cohort were analyzed with the Bioconductor RTCGA package [61] in R. Patient stratification was performed through calculation of the optimal cutpoint for each miRNA signature through the survival cutpoint function while statistical analysis was based on log-rank p-value calculation. KM results were visualized with ggplot in R.

### 4.8. miRNA Expression in Circulating Body Fluids

For the body fluid analysis, miRNA expression data were extracted from the Human miRNA Tissue Atlas database [62]. Dot plots corresponding to VSN miRNA levels in collapsed tissues were created with ggplot in R. Statistical analysis was performed with ANOVA (Holm–Sidak) in SigmaPlot.

## 5. Conclusions and Future Perspectives

This computational study used various transcriptional, diagnostic, and prognostic parameters in order to isolate and evaluate two miRNA signatures with highly predictive power for bladder cancer detection. Τhe significance of our findings lies in both the enhanced diagnostic properties of the reported signatures and their abundance in the body fluids of cancer patients. Furthermore, this study provides bioinformatic evidence regarding the molecular mechanisms that support the functional role and prognostic potential of the same miRNAs. Collectively, our study supports the value of specific miRNA signatures as non-invasive molecular biomarkers for bladder cancer detection and prognosis.

## 6. Patents

No patent currently results from the work reported in this manuscript.

## Data Availability

Data are contained within the article.

## Supplementary Materials

The following supporting information can be downloaded at: https://www.mdpi.com/article/10.3390/ijms242216243/s1.
